# Correction: Chalk Talks for the Clinical Setting: Evaluation of a Medical Education Workshop for Fellows

**DOI:** 10.15766/mep_2374-8265.11441

**Published:** 2024-06-17

## Correction

In the publication “Chalk Talks for the Clinical Setting: Evaluation of a Medical Education Workshop for Fellows,” the third sentence in the second paragraph of the Introduction—

This instructional method makes use of the cognitive theory of multimedia learning whereby educators utilize text and illustrations to explain complex concepts in ways that are understandable and memorable.^6^

—should also have cited the following reference, which should in turn be added to the References list, between current items 6 and 7:

Nelson RE, Richards JB, Ricotta DN. Strategies to elevate whiteboard mini lectures. *Clin Teach.* 2022;19(2):79–85. https://doi.org/10.1111/tct.13479
