# Supplementary material for: Chalk Talks for the Clinical Setting: Evaluation of a Medical Education Workshop for Fellows
Source: MedEdPORTAL. 2024 Mar 5;20:11385. doi: 10.15766/mep_2374-8265.11385 (PMC10912192; doi:10.15766/mep_2374-8265.11385)
Supplement: Supplementary file 1 — Chalk Talk Presentation.pptxAssignment Instructions.docxResources on Creating Chalk Talks.docxFeedback and Evaluation Tool.docxPre- and Postworkshop Survey.docx [file mep_2374-8265.11385-s001.zip › C. Resources on Creating Chalk Talks.docx]

**Appendix C:** Optional Resources on Creating Chalk Talks

*The content in this appendix is optional and can be emailed or distributed to workshop participants after the first didactic session in order to serve as an additional resource to help participants develop and design their chalk talk.*

You may use the following resources as you prepare to develop your chalk talks. These resources are optional.

- Chalk Talks in the Clinical Learning Environment: <https://journals.lww.com/academicmedicine/Fulltext/2023/04000/Chalk_Talks_in_the_Clinical_Learning_Environment.27.aspx>
- Strategies to elevate whiteboard mini lectures:<https://onlinelibrary.wiley.com/doi/abs/10.1111/tct.13479>
- Bringing mini-chalk talks to the bedside to enhance clinical teaching: <https://pubmed.ncbi.nlm.nih.gov/28178911/>
- Twelve tips to make successful medical infographics: <https://pubmed.ncbi.nlm.nih.gov/33342338/>
- [Mentor Chalk Talk Video #1](https://www.youtube.com/watch?v=OIAMau7Oj30&t=276s)
- [Mentor Chalk Talk Video #2](https://www.youtube.com/watch?v=oHBLrPnUY4U&t=14s)
- [Mentor Chalk Talk Video #4](https://www.youtube.com/watch?v=2di3e8hIcwk)
- [Mentor Chalk Talk Video #5](https://www.youtube.com/watch?v=dA5ii2wXnTU&t=404s)
