# Supplementary material for: Chalk Talks for the Clinical Setting: Evaluation of a Medical Education Workshop for Fellows
Source: MedEdPORTAL. 2024 Mar 5;20:11385. doi: 10.15766/mep_2374-8265.11385 (PMC10912192; doi:10.15766/mep_2374-8265.11385)
Supplement: Supplementary file 1 — Chalk Talk Presentation.pptxAssignment Instructions.docxResources on Creating Chalk Talks.docxFeedback and Evaluation Tool.docxPre- and Postworkshop Survey.docx [file mep_2374-8265.11385-s001.zip › D. Feedback and Evaluation Tool.docx]

**Appendix D:** Standardized Chalk Talk Feedback and Evaluation Tool

*This appendix provides a rubric that peers and faculty can use to provide formative feedback to course participants during the chalk talk practice session. Fifty minutes should be allocated for the practice chalk talk session. These sessions should be conducted in smaller rooms with 3 workshop participants and one faculty member per group. Each room must contain a chalkboard or whiteboard. With 3 workshop participants per group, each participant will have 15 minutes to present their chalk talk (5-7 minutes) and receive feedback (6-8 minutes) on their presentation. Workshop participants and faculty should be given this evaluation form prior to the chalk talk practice session so that they can review the rubric and scoring parameters.*

Title of Chalk Talk:

Name of presenter:

Please score the chalk talk based on the following features:

Content Suitability

- 0 = Content is minimally suited for a chalk talk presentation
- 1 = Content is moderately suited for a chalk talk presentation
- 2 = Content is highly suitable for a chalk talk presentation

Chalk delivery

- 0 = Poorly delivered without clear or logical organization or explanation
- 1 = Delivery is mostly clear, although there may be some areas that were confusing
- 2 = Delivery is clear, logical, and effectively achieves learning objectives

Chalk talk design

- 0 = Visuals are absent, confusing, or very difficult to reproduce
- 1 = Visuals provided add to the chalk talk but could be improved for clarity
- 2 = Impactful visuals help clarify content

Learning Objectives

- 0 = No or poorly worded learning objectives
- 1 = Learning objectives are appropriate but could be improved
- 2 = Well-written learning objectives that are specific, measurable, achievable, relevant, and/or time-bound

Active Learning Strategies / Audience Engagement

- 0 = Active learning strategies are absent, confusing, and/or inappropriate for the chalk talk. Chalk talk is minimally interactive.
- 1 = Active learning strategies are used but need some improvement to maximally engage learners. Chalk talk allows for some interactivity.
- 2 = Active learning strategies are appropriate for the chalk talk and provide maximal opportunities for learner engagement.

Length

- 0 = Chalk talk is too long (> 15 minutes)
- 1 = Chalk talk can be delivered within a reasonable amount of time in the clinical setting (5-15 minutes)

Citation: Author Owned
