# Supplementary material for: Chalk Talks for the Clinical Setting: Evaluation of a Medical Education Workshop for Fellows
Source: MedEdPORTAL. 2024 Mar 5;20:11385. doi: 10.15766/mep_2374-8265.11385 (PMC10912192; doi:10.15766/mep_2374-8265.11385)
Supplement: Supplementary file 1 — Chalk Talk Presentation.pptxAssignment Instructions.docxResources on Creating Chalk Talks.docxFeedback and Evaluation Tool.docxPre- and Postworkshop Survey.docx [file mep_2374-8265.11385-s001.zip › E. Pre- and Postworkshop Survey.docx]

**Appendix E**

*The items in this appendix were used to measure self-reported confidence in participants’ ability to create and deliver a chalk talk and write learning objectives. Item 4 measures knowledge about advanced organizers, a foundational concept important for effective chalk talks. Items were administered before the first workshop and after the chalk talk practice session was completed.*

Pre-Workshop Survey

1. Prior to this course, how confident do you feel in your ability to create an effective chalk talk? (Extremely confident, very confident, moderately confident, somewhat confident, not at all confident)
2. Prior to this course, how confident do you feel in your ability to deliver an effective chalk talk? (Extremely confident, very confident, moderately confident, somewhat confident, not at all confident)
3. Prior to this course, how confident do you feel in your ability to develop smart, measurable, attainable, relevant, and time-bound learning objectives? (Extremely confident, very confident, moderately confident, somewhat confident, not at all confident)
4.
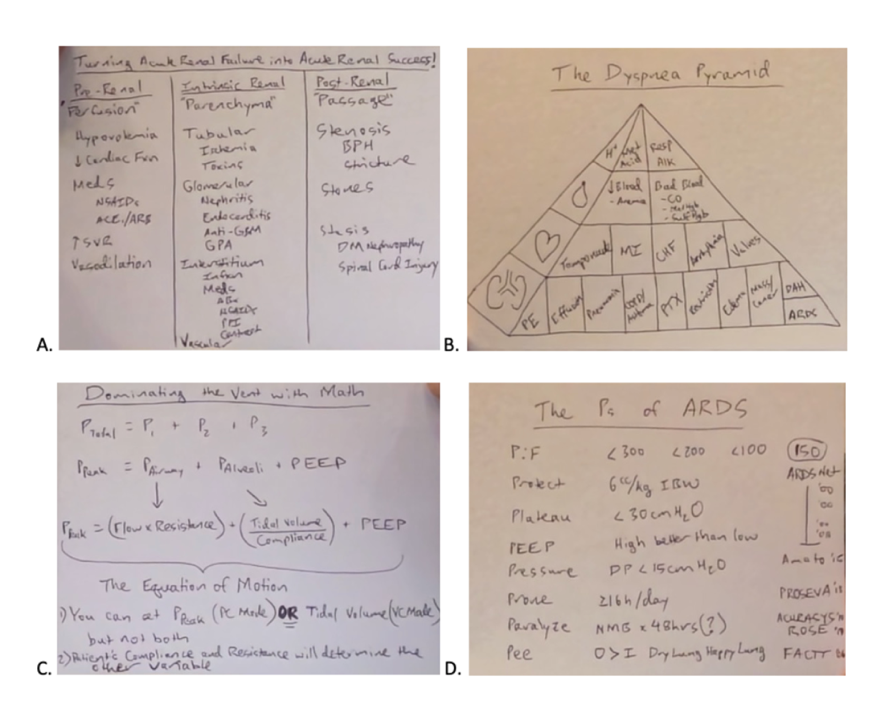
Which of the following chalk talks makes use of an advance organizer?

Post-Workshop Survey

1. Because of what you learned in this course, how confident do you feel in your ability to create an effective chalk talk? (Extremely confident, very confident, moderately confident, somewhat confident, not at all confident)
2. Because of what you learned in this course, how confident do you feel in your ability to deliver an effective chalk talk? (Extremely confident, very confident, moderately confident, somewhat confident, not at all confident)
3. Because of what you learned in this course, how confident do you feel in your ability to develop smart, measurable, attainable, relevant, and time-bound learning objectives? (Extremely confident, very confident, moderately confident, somewhat confident, not at all confident)
4.
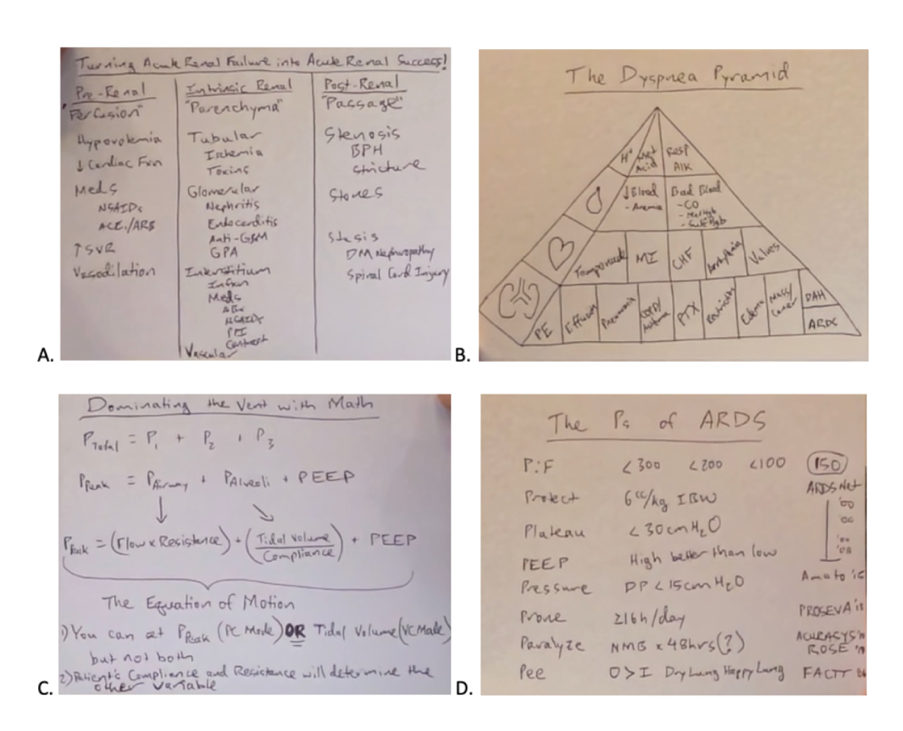
Which of the following chalk talks makes use of an advance organizer?

Citation: Author Owned
