# Supplementary material for: Chalk Talks for the Clinical Setting: Evaluation of a Medical Education Workshop for Fellows
Source: MedEdPORTAL. 2024 Mar 5;20:11385. doi: 10.15766/mep_2374-8265.11385 (PMC10912192; doi:10.15766/mep_2374-8265.11385)
Supplement: Supplementary file 1 — Chalk Talk Presentation.pptxAssignment Instructions.docxResources on Creating Chalk Talks.docxFeedback and Evaluation Tool.docxPre- and Postworkshop Survey.docx [file mep_2374-8265.11385-s001.zip › A. Chalk Talk Presentation.pptx]

## Slide 1
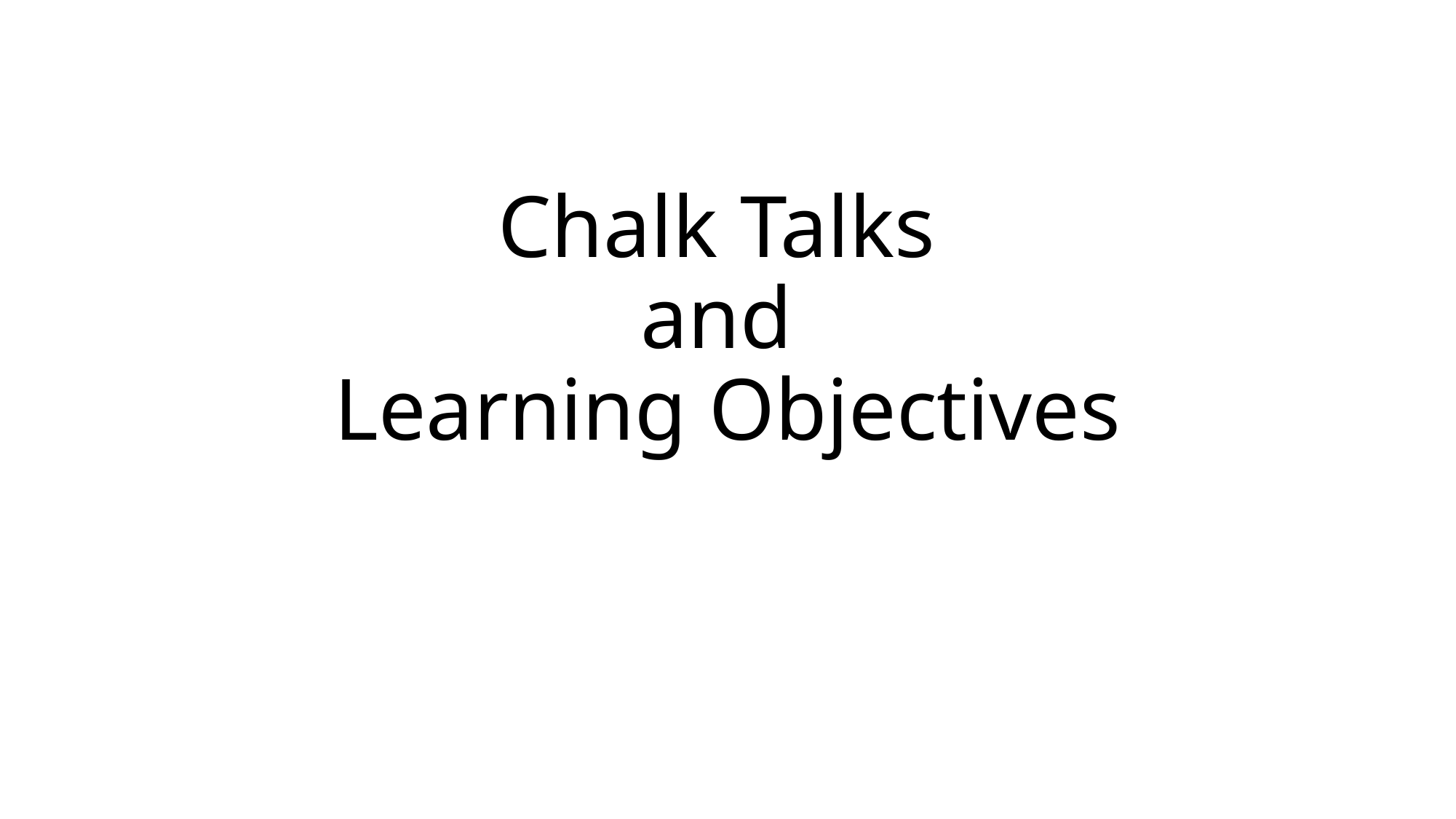

# Chalk Talks and Learning Objectives

## Slide 2
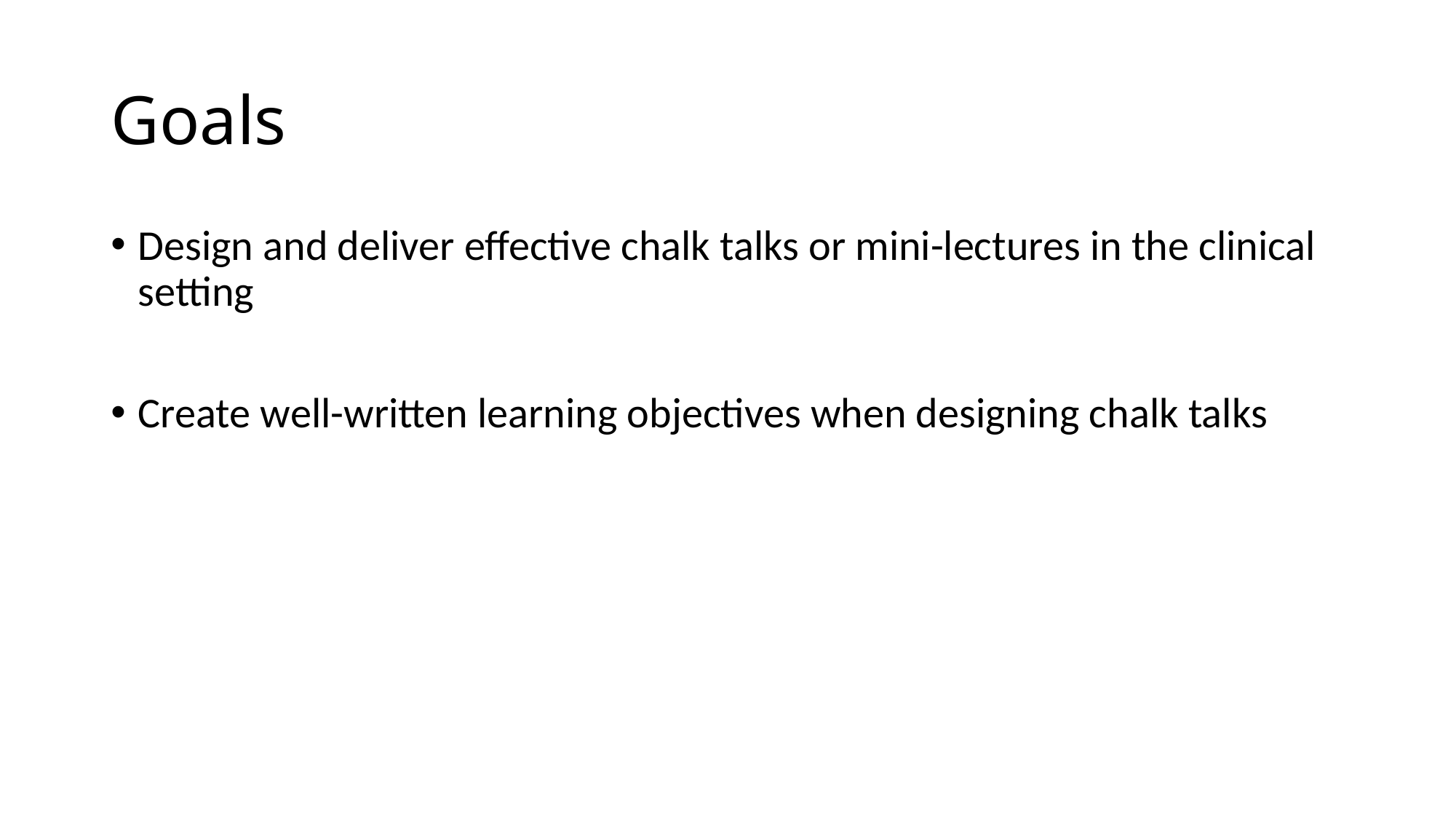

# Goals
Design and deliver effective chalk talks or mini-lectures in the clinical setting
Create well-written learning objectives when designing chalk talks

## Slide 3
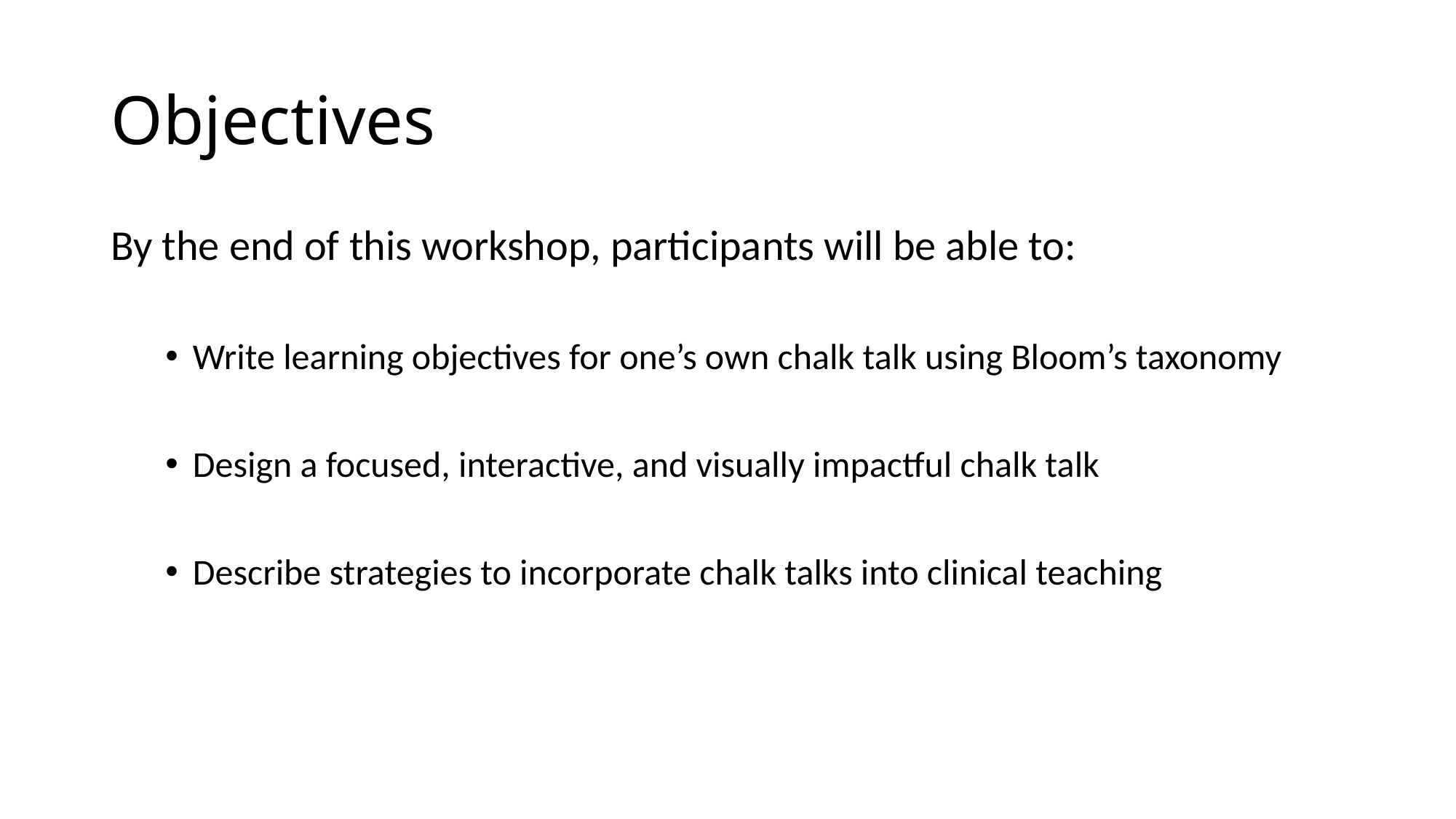

# Objectives
By the end of this workshop, participants will be able to:
Write learning objectives for one’s own chalk talk using Bloom’s taxonomy
Design a focused, interactive, and visually impactful chalk talk
Describe strategies to incorporate chalk talks into clinical teaching

## Slide 4
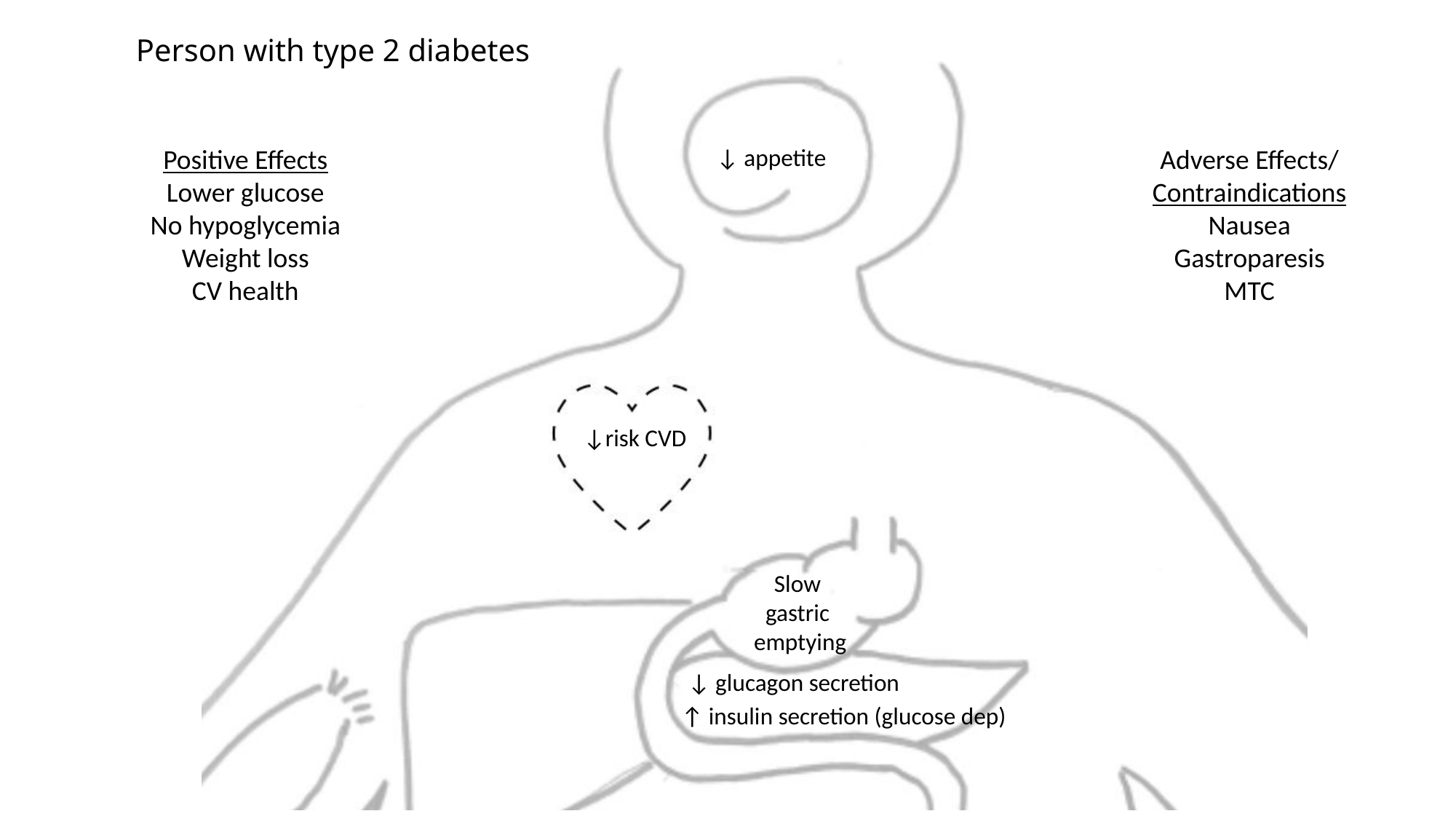

Person with type 2 diabetes
↓ appetite
Adverse Effects/
Contraindications
Nausea
Gastroparesis
MTC
Positive Effects
Lower glucose
No hypoglycemia
Weight loss
CV health
↓risk CVD
Slow
gastric
emptying
↓ glucagon secretion
↑ insulin secretion (glucose dep)

## Slide 5
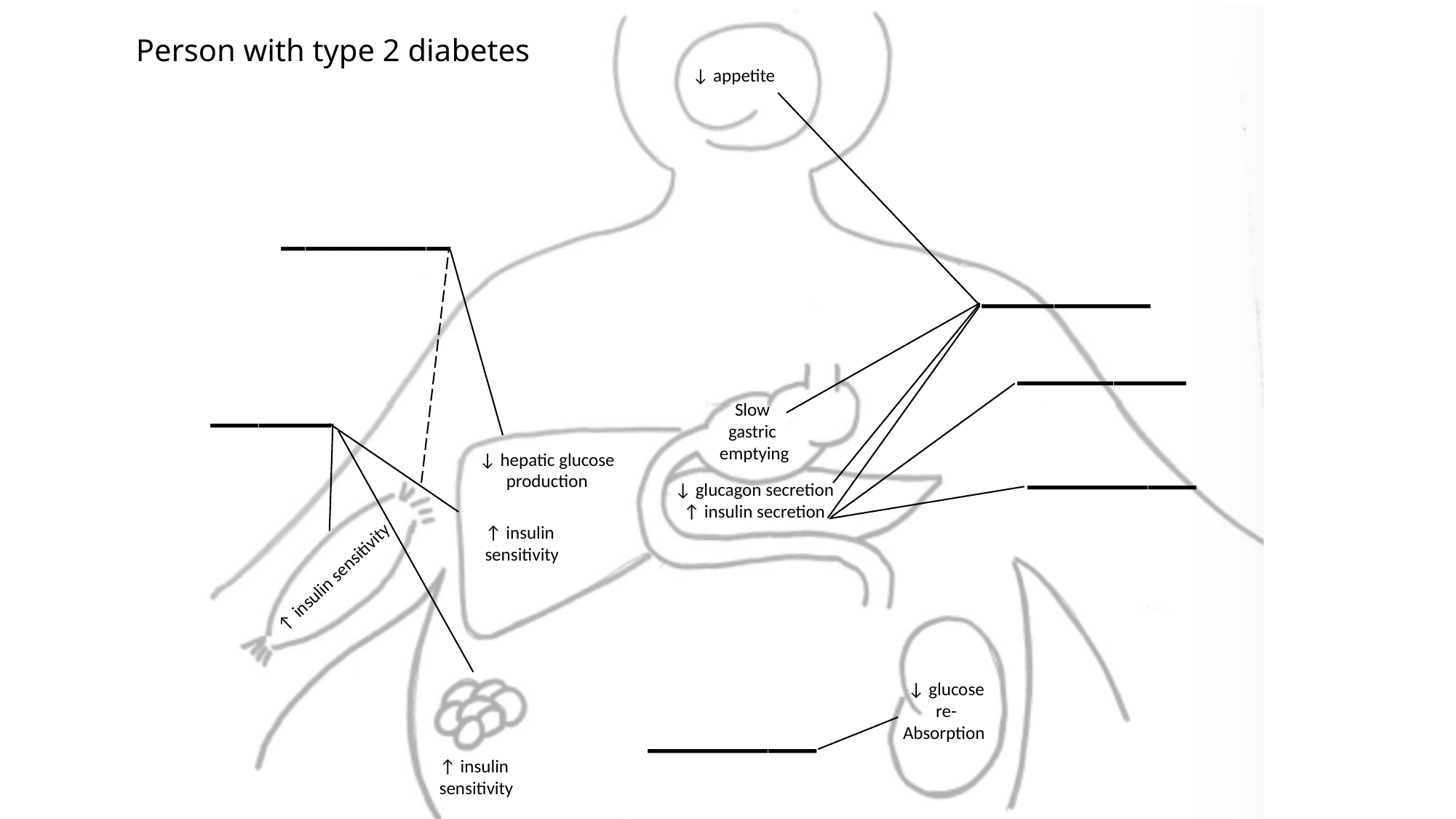

Person with type 2 diabetes
↓ appetite
_______
_______
_______
_____
Slow
gastric
emptying
_______
↓ hepatic glucose production
↓ glucagon secretion
↑ insulin secretion
↑ insulin
sensitivity
↑ insulin sensitivity
↓ glucose
re-
Absorption
_______
↑ insulin
sensitivity

## Slide 6
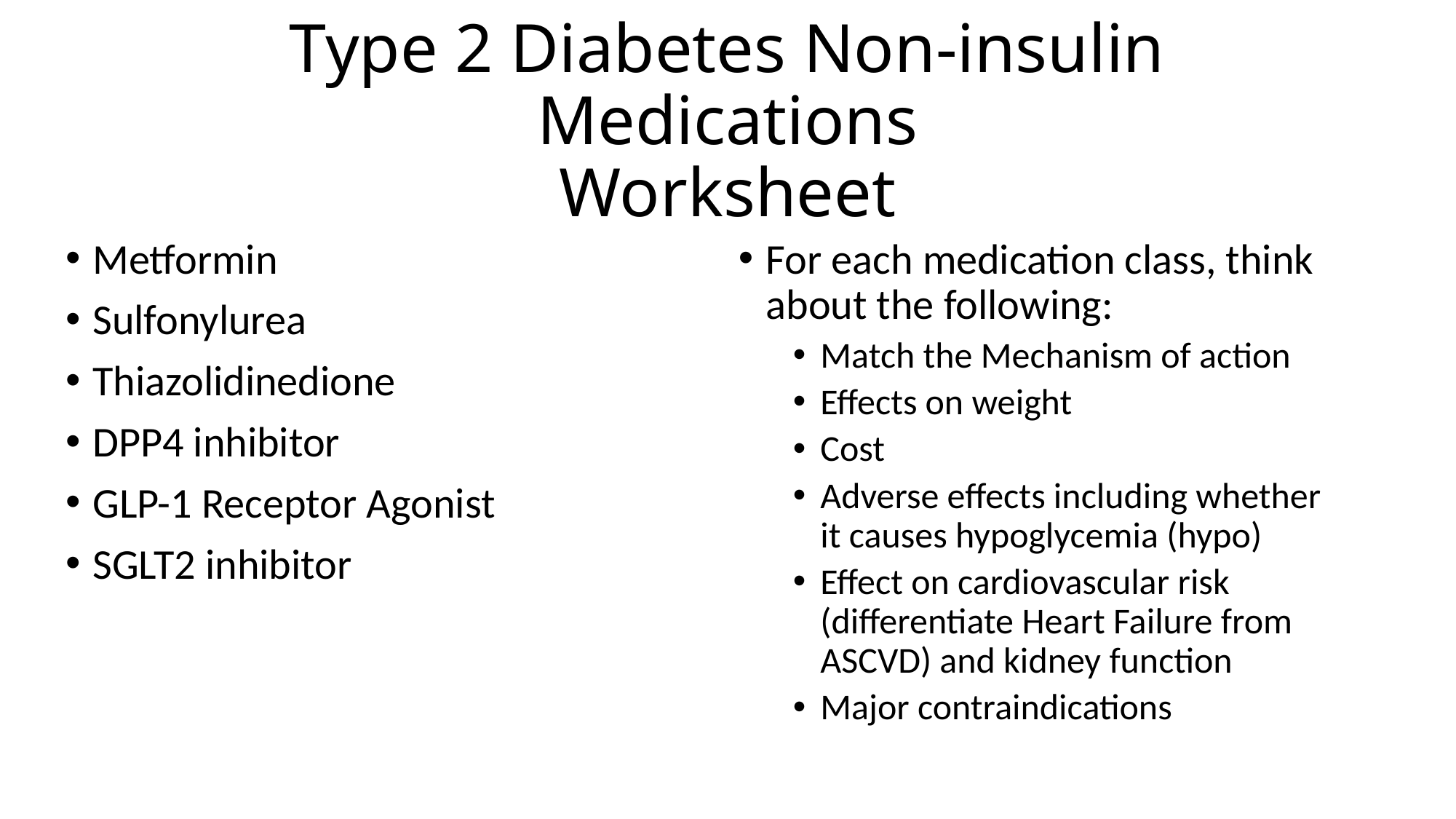

# Type 2 Diabetes Non-insulin Medications Worksheet
Metformin
Sulfonylurea
Thiazolidinedione
DPP4 inhibitor
GLP-1 Receptor Agonist
SGLT2 inhibitor
For each medication class, think about the following:
Match the Mechanism of action
Effects on weight
Cost
Adverse effects including whether it causes hypoglycemia (hypo)
Effect on cardiovascular risk (differentiate Heart Failure from ASCVD) and kidney function
Major contraindications

## Slide 7
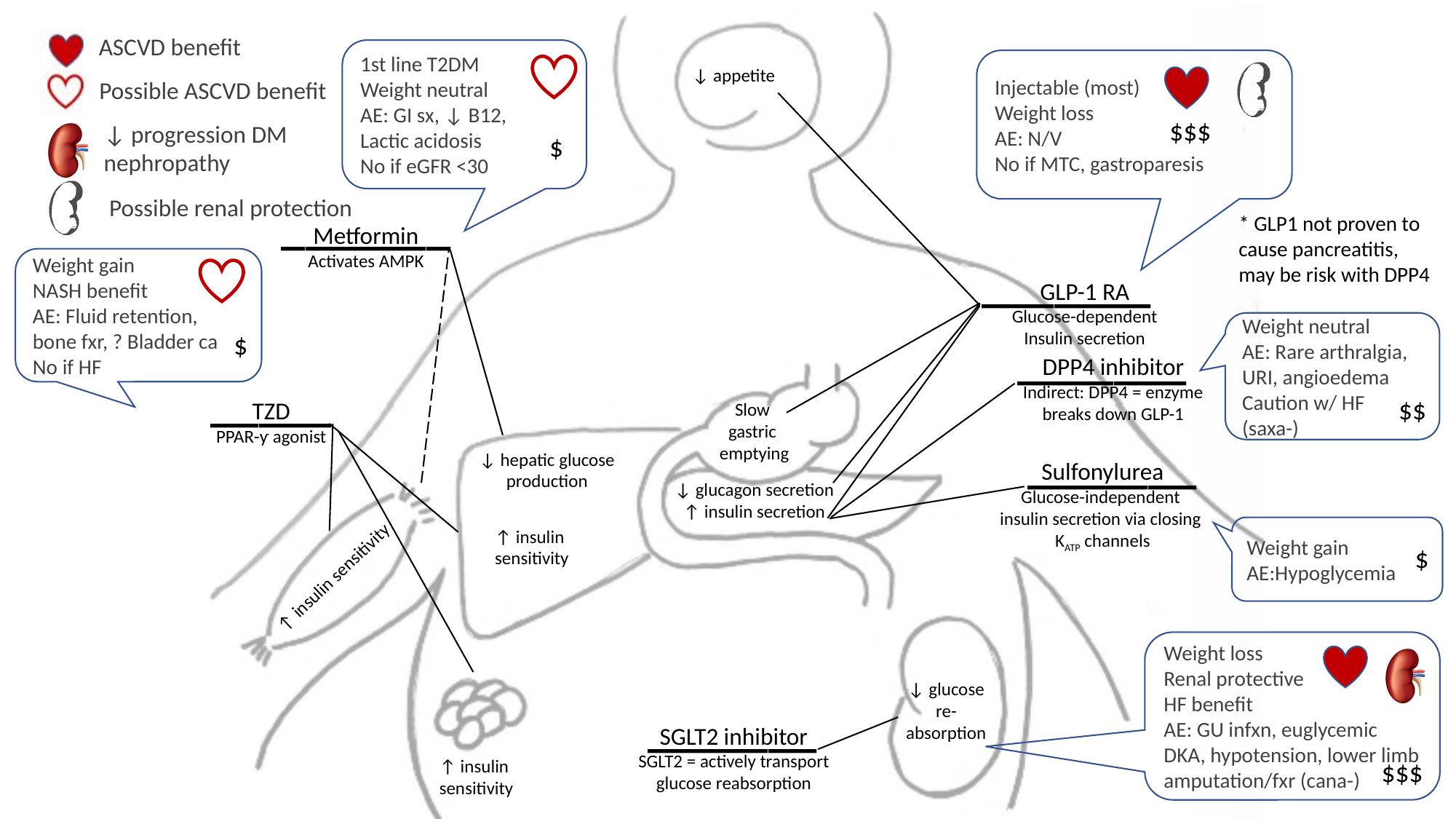

ASCVD benefit
1st line T2DM
Weight neutral
AE: GI sx, ↓ B12, Lactic acidosis
No if eGFR <30
Injectable (most)
Weight loss
AE: N/V
No if MTC, gastroparesis
↓ appetite
Possible ASCVD benefit
$$$
↓ progression DM nephropathy
$
Possible renal protection
_______
* GLP1 not proven to cause pancreatitis, may be risk with DPP4
Metformin
Activates AMPK
_______
Weight gain
NASH benefit
AE: Fluid retention, bone fxr, ? Bladder ca
No if HF
GLP-1 RA
Glucose-dependent
Insulin secretion
Weight neutral
AE: Rare arthralgia, URI, angioedema
Caution w/ HF
(saxa-)
$
_______
DPP4 inhibitor
Indirect: DPP4 = enzyme breaks down GLP-1
_____
$$
TZD
PPAR-ƴ agonist
Slow
gastric
emptying
_______
↓ hepatic glucose production
Sulfonylurea
Glucose-independent
insulin secretion via closing
KATP channels
↓ glucagon secretion
↑ insulin secretion
Weight gain
AE:Hypoglycemia
↑ insulin
sensitivity
$
↑ insulin sensitivity
Weight loss
Renal protective
HF benefit
AE: GU infxn, euglycemic DKA, hypotension, lower limb amputation/fxr (cana-)
↓ glucose
re-
absorption
_______
SGLT2 inhibitor
SGLT2 = actively transport glucose reabsorption
↑ insulin
sensitivity
$$$

## Slide 8
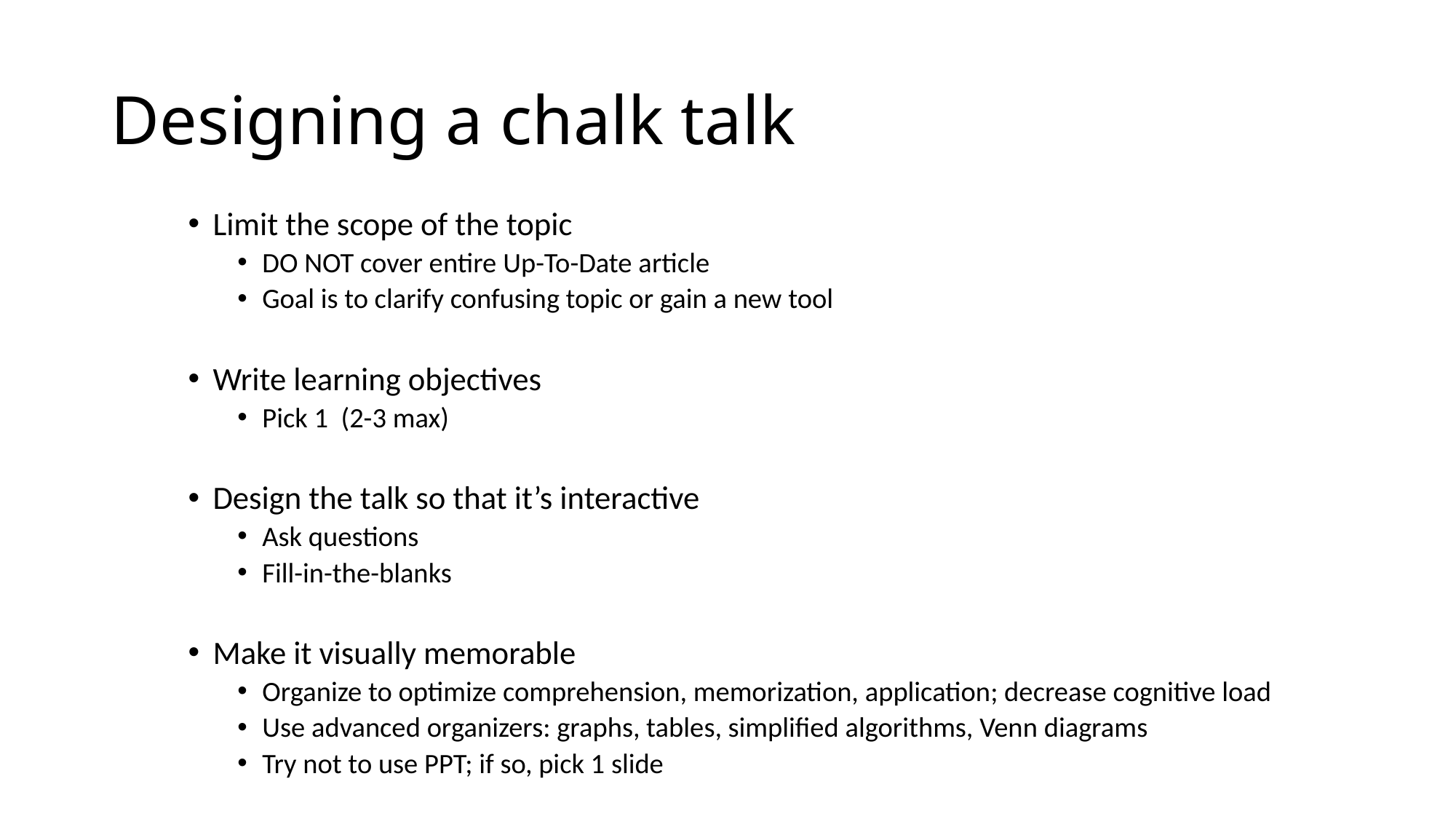

# Designing a chalk talk
Limit the scope of the topic
DO NOT cover entire Up-To-Date article
Goal is to clarify confusing topic or gain a new tool
Write learning objectives
Pick 1 (2-3 max)
Design the talk so that it’s interactive
Ask questions
Fill-in-the-blanks
Make it visually memorable
Organize to optimize comprehension, memorization, application; decrease cognitive load
Use advanced organizers: graphs, tables, simplified algorithms, Venn diagrams
Try not to use PPT; if so, pick 1 slide

## Slide 9
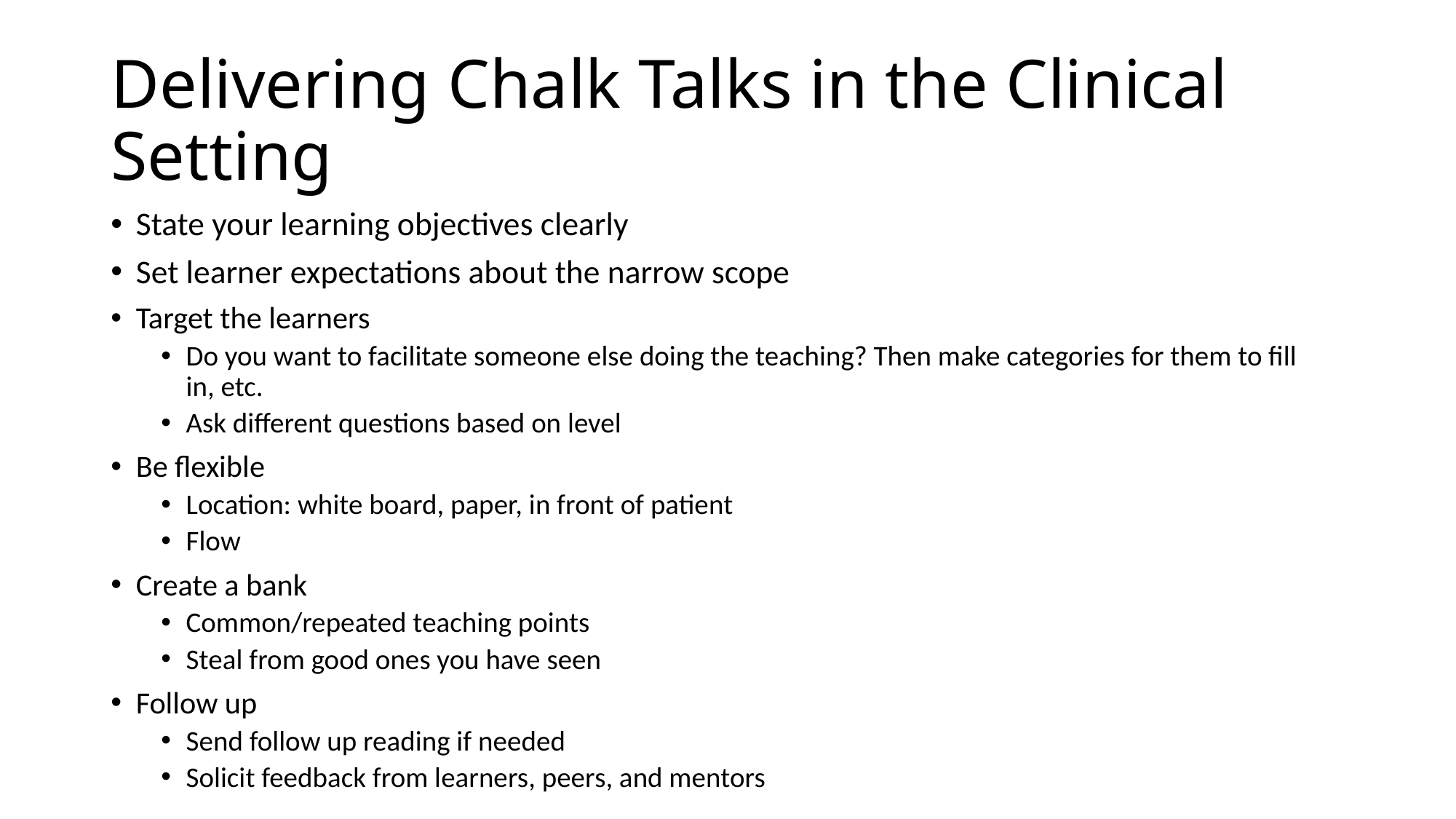

# Delivering Chalk Talks in the Clinical Setting
State your learning objectives clearly
Set learner expectations about the narrow scope
Target the learners
Do you want to facilitate someone else doing the teaching? Then make categories for them to fill in, etc.
Ask different questions based on level
Be flexible
Location: white board, paper, in front of patient
Flow
Create a bank
Common/repeated teaching points
Steal from good ones you have seen
Follow up
Send follow up reading if needed
Solicit feedback from learners, peers, and mentors

## Slide 10
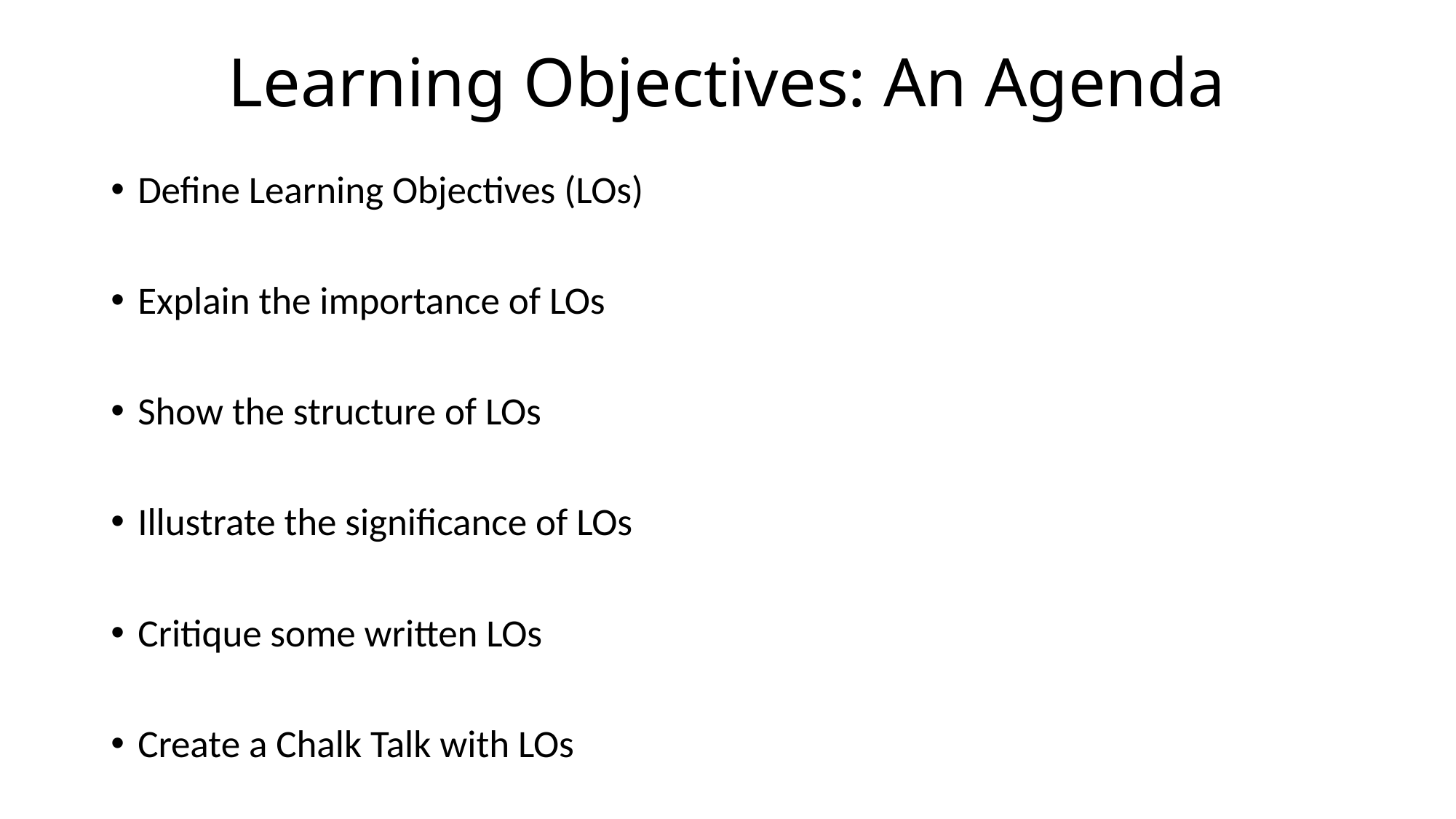

# Learning Objectives: An Agenda
Define Learning Objectives (LOs)
Explain the importance of LOs
Show the structure of LOs
Illustrate the significance of LOs
Critique some written LOs
Create a Chalk Talk with LOs

## Slide 11
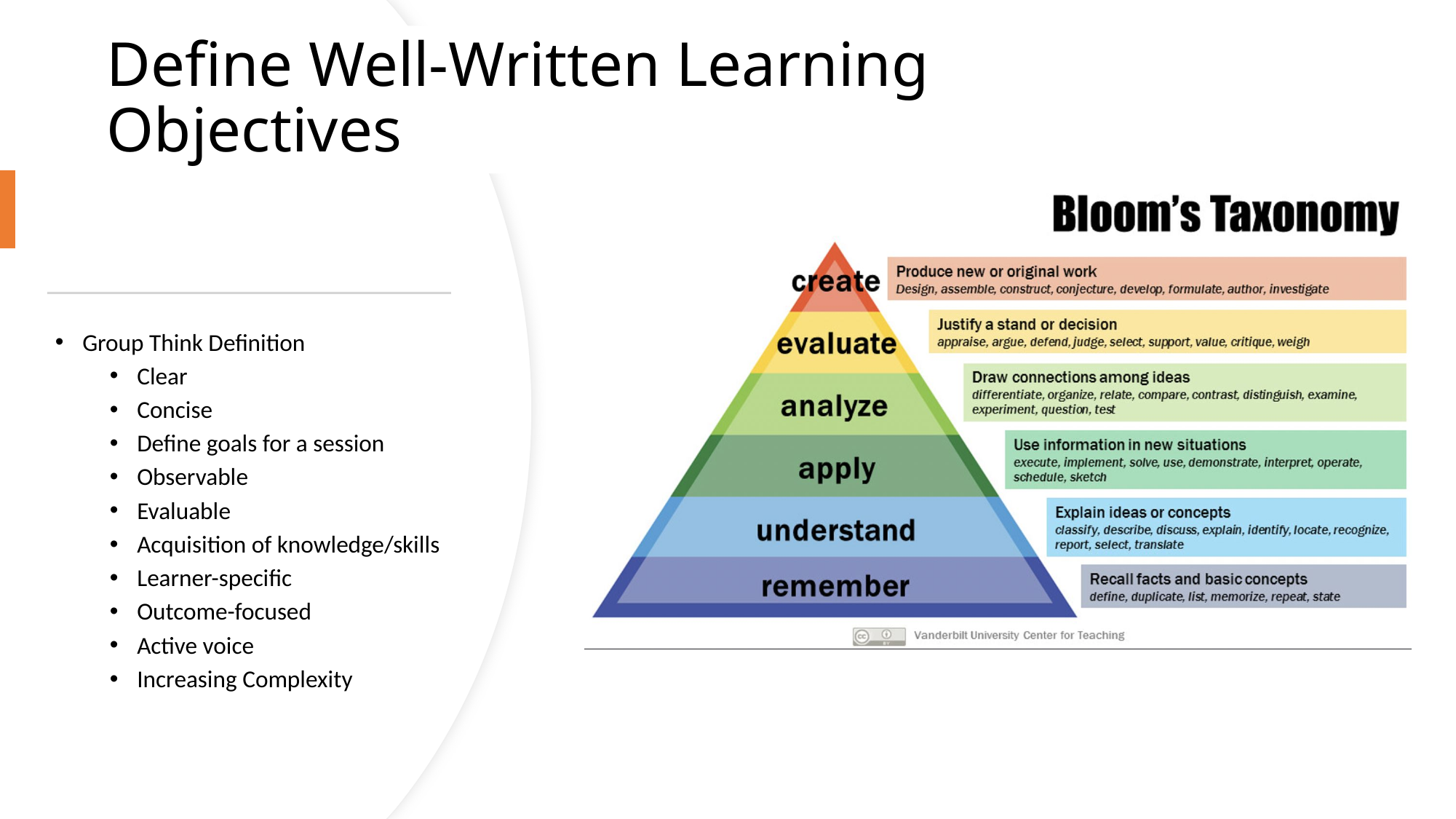

# Define Well-Written Learning Objectives
Group Think Definition
Clear
Concise
Define goals for a session
Observable
Evaluable
Acquisition of knowledge/skills
Learner-specific
Outcome-focused
Active voice
Increasing Complexity

## Slide 12
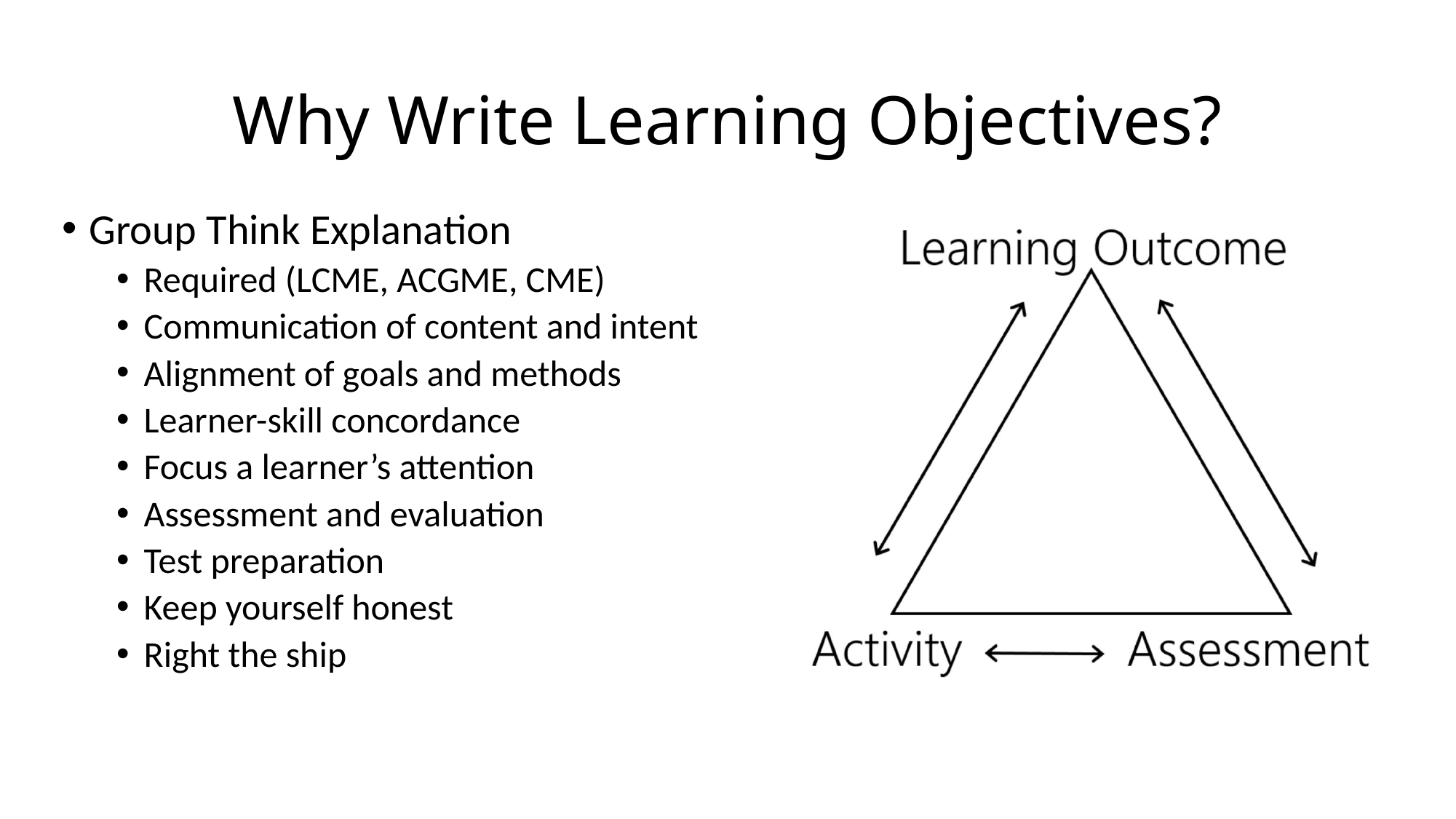

# Why Write Learning Objectives?
Group Think Explanation
Required (LCME, ACGME, CME)
Communication of content and intent
Alignment of goals and methods
Learner-skill concordance
Focus a learner’s attention
Assessment and evaluation
Test preparation
Keep yourself honest
Right the ship

## Slide 13
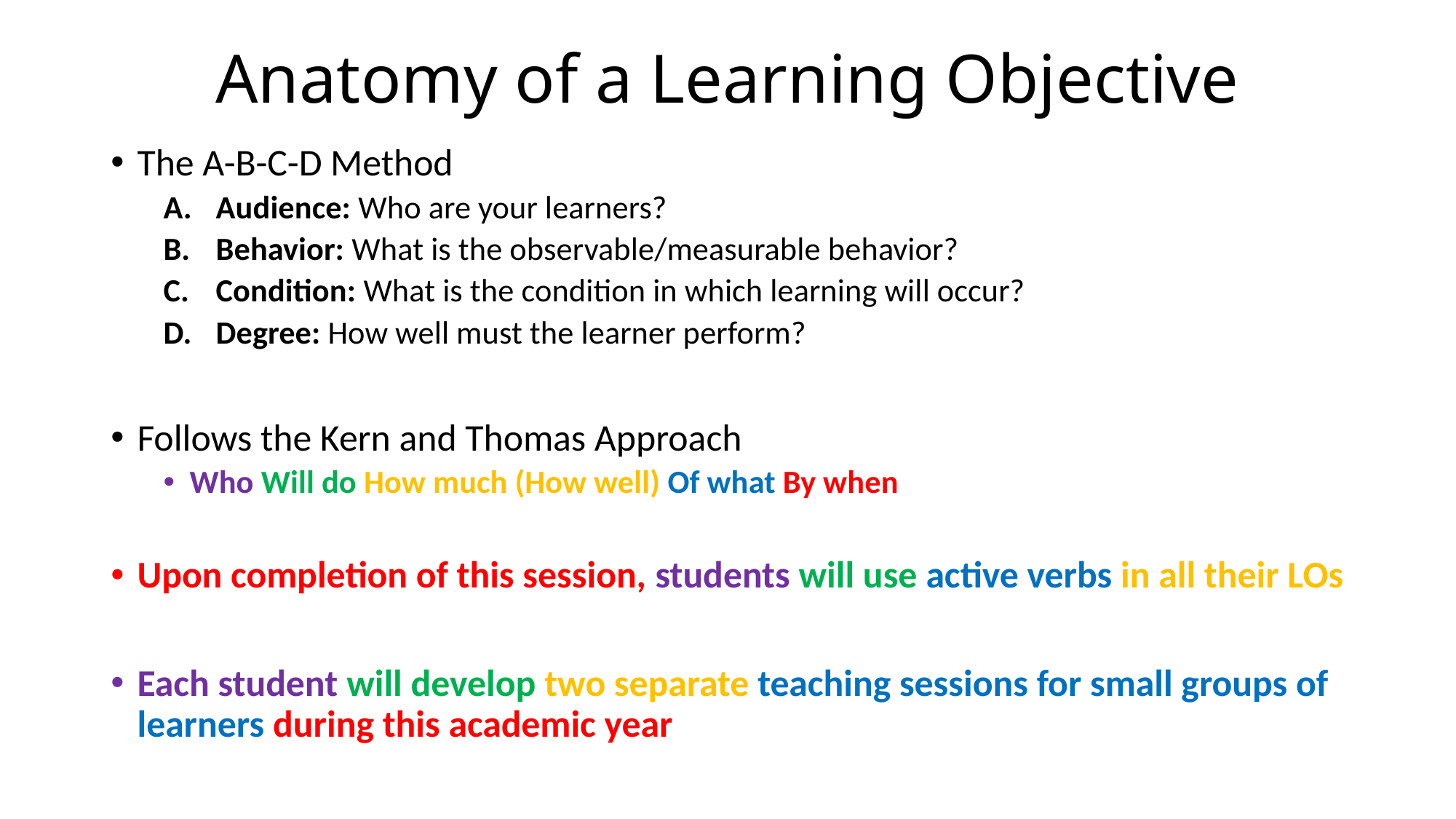

# Anatomy of a Learning Objective
The A-B-C-D Method
Audience: Who are your learners?
Behavior: What is the observable/measurable behavior?
Condition: What is the condition in which learning will occur?
Degree: How well must the learner perform?
Follows the Kern and Thomas Approach
Who Will do How much (How well) Of what By when
Upon completion of this session, students will use active verbs in all their LOs
Each student will develop two separate teaching sessions for small groups of learners during this academic year

## Slide 14
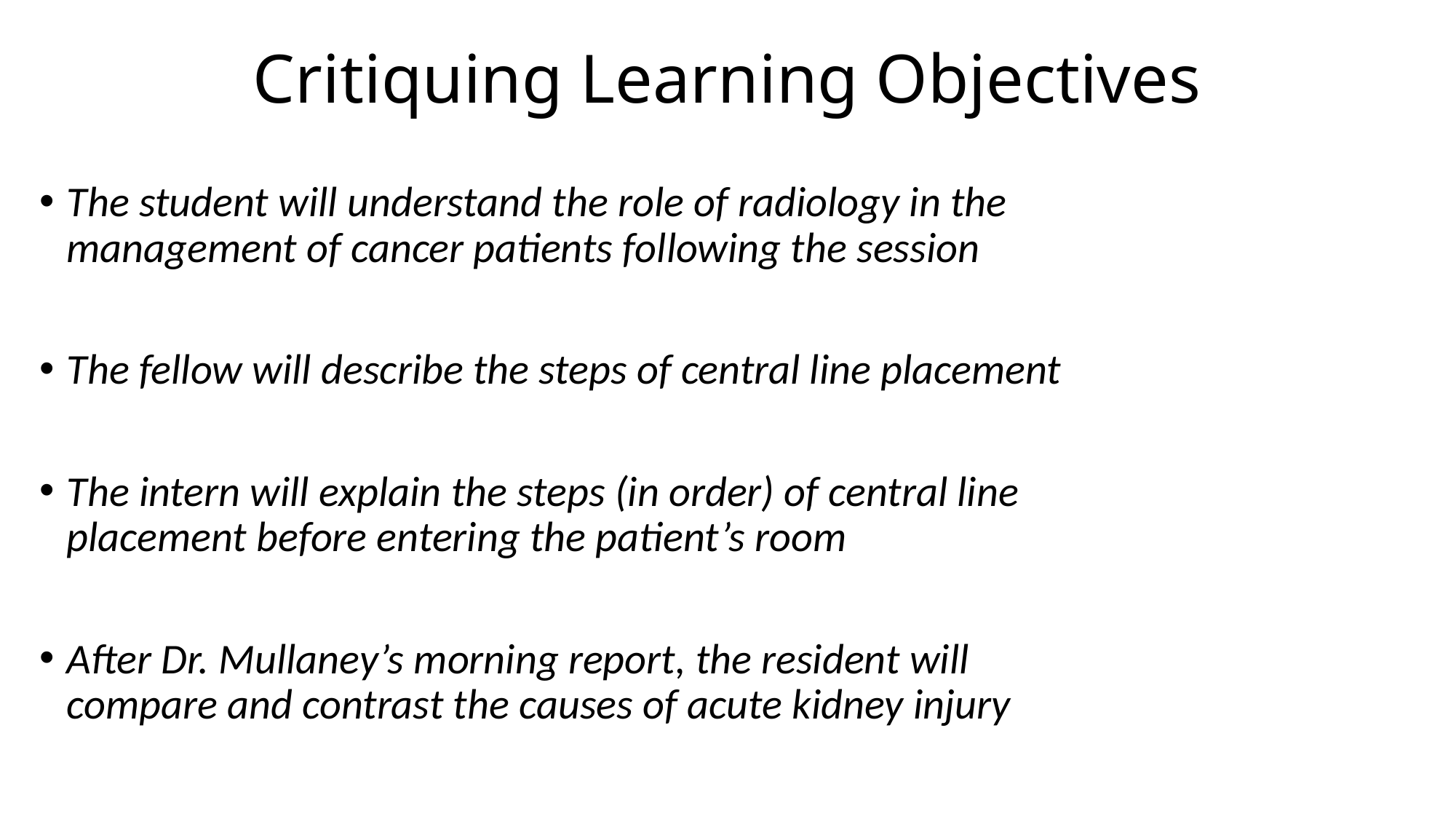

# Critiquing Learning Objectives
The student will understand the role of radiology in the management of cancer patients following the session
The fellow will describe the steps of central line placement
The intern will explain the steps (in order) of central line placement before entering the patient’s room
After Dr. Mullaney’s morning report, the resident will compare and contrast the causes of acute kidney injury

## Slide 15
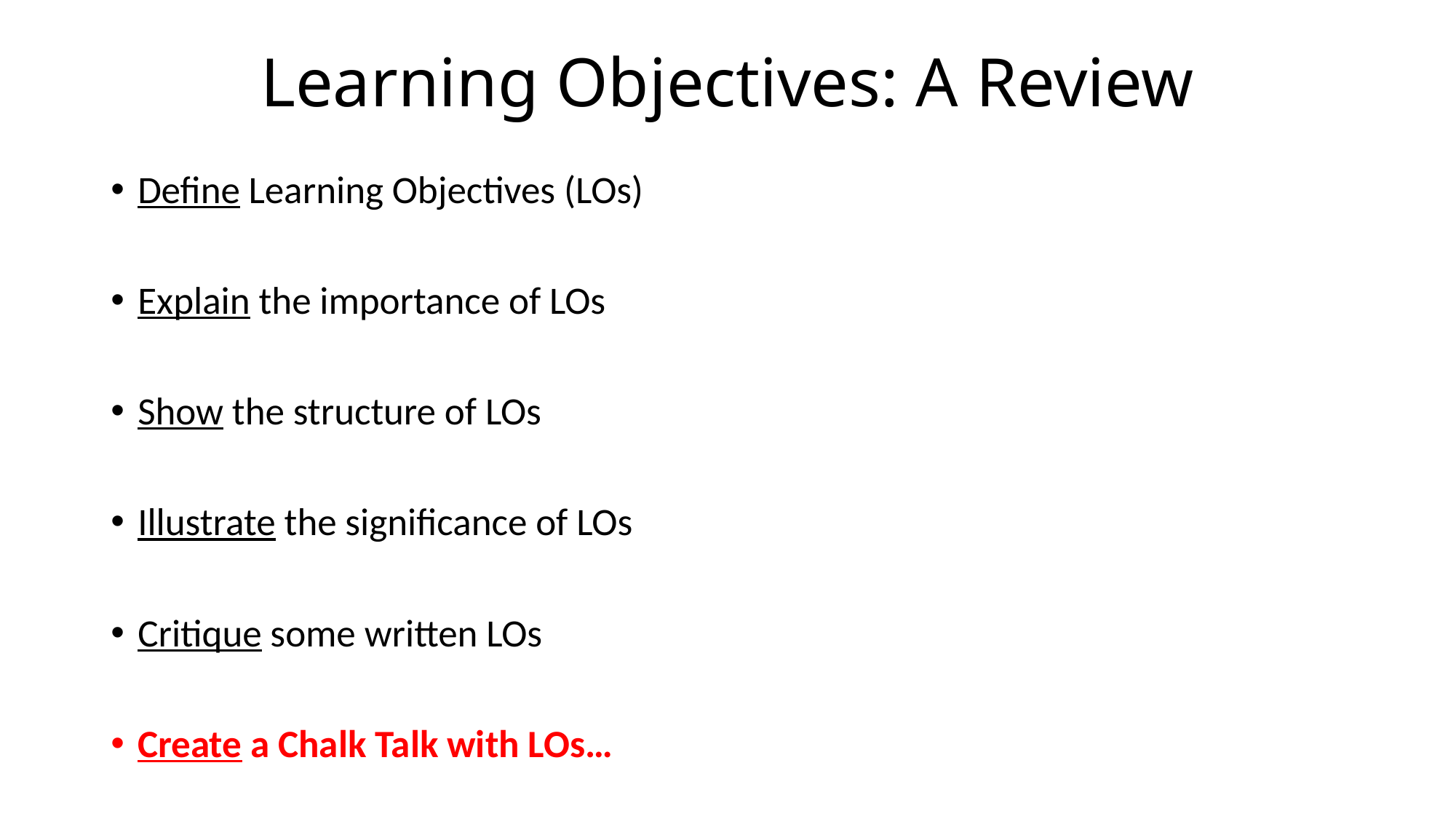

# Learning Objectives: A Review
Define Learning Objectives (LOs)
Explain the importance of LOs
Show the structure of LOs
Illustrate the significance of LOs
Critique some written LOs
Create a Chalk Talk with LOs…
