# Supplementary material for: Chalk Talks for the Clinical Setting: Evaluation of a Medical Education Workshop for Fellows
Source: MedEdPORTAL. 2024 Mar 5;20:11385. doi: 10.15766/mep_2374-8265.11385 (PMC10912192; doi:10.15766/mep_2374-8265.11385)
Supplement: Supplementary file 1 — Chalk Talk Presentation.pptxAssignment Instructions.docxResources on Creating Chalk Talks.docxFeedback and Evaluation Tool.docxPre- and Postworkshop Survey.docx [file mep_2374-8265.11385-s001.zip › B. Assignment Instructions.docx]

**Appendix B:** Assignment Instructions on Creating Chalk Talks

*The content in this appendix can be emailed or distributed to workshop participants after the first didactic session to provide guidance on how to develop and prepare their chalk talk for the practice chalk talk session.*

Please use the following guidance as you prepare and create your chalk talk. You will present your chalk talk to a small group of peers and faculty. Your peers and faculty will provide you with structured feedback using the “Standardized Chalk Talk Feedback and Evaluation Tool.”

- Choose a topic in your specialty that you are likely to use in real life.
- Focus your topic on something that can be covered in less than 5 minutes.
- Write 1-2 learning objectives using the framework provided in class; be sure to explicitly state your learning objectives at the beginning when you deliver your chalk talk.
  - Write your learning objectives for your future (in real life) learners; you do not need to prepare learning objectives catered to your peers (who might not be familiar with your specialty)
- Strategize ways in which you will engage your audience when you deliver the chalk talk.
- Practice (practice, practice) your talk prior to your chalk talk presentation.
- You can use notes when delivering your chalk talk.
- You will have 2-3 different colors you can use during your chalk talk (whiteboard markers).
- Remember that less is more.
