# Supplementary material for: Chalk Talks for the Clinical Setting: Evaluation of a Medical Education Workshop for Fellows
Source: MedEdPORTAL. 2024 Mar 5;20:11385. doi: 10.15766/mep_2374-8265.11385 (PMC10912192; doi:10.15766/mep_2374-8265.11385)
Supplement: Supplementary file 1 — Chalk Talk Presentation.pptxAssignment Instructions.docxResources on Creating Chalk Talks.docxFeedback and Evaluation Tool.docxPre- and Postworkshop Survey.docx [file mep_2374-8265.11385-s001.zip › _Educational Summary Report_11385.pdf]

MedEdPORTAL® The AAMC Journal of Teaching and Learning Resources

Original Publication Open Access

Chalk Talks for the Clinical Setting: Evaluation of a Medical Education Workshop for Fellows

Shady I. Soliman, MD, MS, William McGuire, MD, Tricia Santos, MD, Charlie Goldberg, MD, MS, Charles Coffey, MD, Darcy Wooten, MD, MS*

*Corresponding author: dawooten@health.ucsd.edu

Abstract

Introduction: Chalk talks are effective teaching tools in the clinical setting. However, data on optimal strategies for teaching medical educators how to develop and deliver them are limited. We designed and implemented two 50-minute workshops to help subspecialty fellows across GME create and deliver a chalk talk. **Methods:** The first workshop comprised a demonstration of an effective chalk talk and a discussion of best practices for creating chalk talks; the second was a practice session where fellows presented their chalk talks and received feedback from faculty and peers. We evaluated pre- and postworkshop confidence in the ability to create and deliver a chalk talk and develop learning objectives. Secondary outcomes were faculty and peer evaluations of the chalk talks. **Results:** Eighteen of 33 participants (54% response rate) completed both pre- and postsession surveys. Fellows reported improved confidence in their ability to create a chalk talk (22% vs. 83%, $p < .001$), deliver a chalk talk (17% vs. 83%, $p < .001$), and develop well-written learning objectives (11% vs. 83%, $p < .001$). After the workshop, participants were more likely to correctly identify a chalk talk that made use of an advanced organizer (67% vs. 89%, $p < .05$). Thirty-eight faculty and peers completed feedback evaluations of participants' chalk talks; most rated fellows' chalk talks highly in domains of content, delivery, design, learning objectives, and engagement. **Discussion:** The incorporation of these workshop within a course on medical education can effectively develop clinical teaching skills among subspecialty fellows in GME.

Keywords
Chalk Talks, Learning Objectives, Clinical Teaching/Bedside Teaching, Multimedia

Educational Objectives

By the end of this two-part workshop, participants will be able to:

1. Write learning objectives for their own chalk talk using Bloom's taxonomy.
2. Design a focused, interactive, and visually impactful chalk talk.
3. Describe strategies to incorporate chalk talks into clinical teaching.

Introduction

Teaching trainees to become effective educators in the clinical setting is an integral goal of subspecialty fellowship training.[1] After training, fellows often remain in academia where they are responsible for teaching a variety of learners (students, residents, fellows) and are increasingly pursuing clinician-educator careers.[2] Nonetheless, fellows predominantly develop clinical teaching skills on the job without intentional curricula or deliberate practice, and many report a lack of formal programs to advance their teaching skills.[3]

Teaching in the clinical setting is challenging for several reasons, including time constraints, prioritization of patient care, and requirements for frequent multitasking.[4,5] Chalk talks are small-group, interactive didactics that utilize a chalkboard or whiteboard. This instructional method makes use of the cognitive theory of multimedia learning whereby educators utilize text and illustrations to explain complex concepts in ways that are understandable and memorable.[6] There are several proposed benefits of chalk talks in the clinical teaching environment, such as improved engagement and interest, real-time interaction with the material, and an understanding of a manageable amount of content through reductions in cognitive load.[7] Chalk talks can also provide a visual framework that allows for meaningful clinical teaching prior to, during, or

Citation:
Soliman SI, McGuire W, Santos T, Goldberg C, Coffey C, Wooten D. Chalk talks for the clinical setting: evaluation of a medical education workshop for fellows. *MedEdPORTAL*. 2024;20:11385.
https://doi.org/10.15766/mep_2374-8265.11385

Copyright © 2024 Soliman et al. This is an open-access publication distributed under the terms of the Creative Commons Attribution license.

after patient encounters. Importantly, chalk talks may allow learners to navigate the conceptual framework of the presenter's clinical knowledge and reasoning by focusing on concrete learning objectives.

Chalk talks represent a high-yield clinical teaching method,[6,8,9] but published data on the most effective strategies to teach educators how to develop and deliver chalk talks in the clinical setting are limited. Additionally, no curricula on clinical teaching skills published in *MedEdPORTAL* focus exclusively on chalk talks. To address these gaps, we developed two complimentary workshops that included both a didactic component focused on foundational principles for creating a chalk talk and deliberate practice of chalk talk delivery with structured feedback and evaluations. Our learner audience centered on subspecialty fellows across GME at the University of California, San Diego (UCSD).

We evaluated the impact of the chalk talk didactic and deliberate practice on fellows' self-reported confidence in their ability to design and deliver a chalk talk in the clinical setting and their change in knowledge of medical education principles important for designing chalk talks before compared to after the workshops. We also assessed formative feedback provided by peers and faculty on fellows' delivery of their chalk talks during a practice session.

Methods

Curriculum

The chalk talk workshops consisted of three components: (1) a 50-minute didactic session focused on how to design and deliver chalk talks in the clinical setting, (2) an assignment for fellows to create a unique chalk talk on a topic of their choice, and (3) a 50-minute small-group practice session in which fellows delivered their talks to peers and faculty, who used a standardized tool to provide formative feedback. Fellows had 2 weeks between the didactic and the chalk talk practice session to design their talks.

The goals of the 50-minute didactic session (Appendix A) were to review the principles of an effective chalk talk, including writing learning objectives based on Bloom's taxonomy,[10] and to examine the elements of an effective chalk talk. During the session, the instructor first delivered a chalk talk on the mechanism of GLP-1 receptor agonists for the treatment of diabetes, discussing and modeling audience engagement, the use of advanced organizers (tools that arrange text and graphics in a way that enhances learners' understanding of the material and links information to knowledge that learners already have in a generative way), and a focused scope of content directly related to the chalk talk's learning objectives. The second half of the didactic taught learners how to write learning objectives using Bloom's taxonomy.

Following the didactic, fellows were provided with instructions on how to create their chalk talks (Appendix B), additional articles and videos on creating effective chalk talks for review outside of class (Appendix C), and a standardized evaluation tool used to offer formative feedback to fellows during the practice session (Appendix D).

For the 50-minute chalk talk practice session, fellows delivered their talks to two other fellows and one faculty member. Each fellow had 5-7 minutes to present their talk, followed by 8-10 minutes of verbal feedback. During the practice session, peers and faculty completed the standardized formative evaluation form (Appendix D); the aggregated results were given to each fellow to review after the session. The faculty reviewers were clinician educators from the UCSD School of Medicine who spanned disciplines (internal medicine, psychiatry, otolaryngology, emergency medicine, etc.) and had varying degrees of formal training in medical education. Faculty reviewers received the evaluation form prior to the session to familiarize themselves with the scoring rubric.

Curricular Context

The chalk talk workshops took place within a course on medical education and clinical teaching for subspecialty fellows at UCSD. The course included four 4-hour didactic sessions that took place weekly for 1 month. During the remainder of the academic year, fellows participated in hands-on teaching experiences with medical students and residents and completed a longitudinal scholarly project. In addition to chalk talks, the course also covered the following content: how to give an effective lecture, facilitating small-group learning, teaching on the wards, teaching in clinic, teaching procedures, simulation in medical education, communication skills, leadership skills, feedback in the clinical setting, critical appraisal of the medical literature, curriculum development, adult learning theories, medical education scholarship, and achieving success and academic advancement as a clinician educator.

Learner Audience

All fellows in GME subspecialty training programs at UCSD and Rady Children's Hospital were eligible to participate in the course. During the 2022-2023 course, 33 fellows across 16 subspecialities were enrolled.

Copyright © 2024 Soliman et al. This is an open-access publication distributed under the terms of the Creative Commons Attribution license.

Workshop Evaluation

We assessed the impact of the chalk talk workshops on fellows who participated in the course during the 2022-2023 academic year. We surveyed fellows before and after the workshops to measure their self-reported confidence in creating and delivering a chalk talk as well as their confidence in writing well-written learning objectives using Bloom's taxonomy (Appendix E). Survey questions were constructed on a 5-point Likert scale (1 = *Not Confident at All,* 5 = *Extremely Confident*). Using multiple-choice questions, we also measured fellows' knowledge of advanced organizers in chalk talk design and identification of well-written learning objectives.

Surveys were developed using the recommendations and foundational principles as outlined by Artino and colleagues.[11] Pre- and postworkshop surveys were identifiable, which enabled evaluations to be associated with specific participants. We also reviewed the scores on the formative feedback forms completed by peers and faculty for each chalk talk delivered during the practice session (Appendix D).

All surveys were developed digitally (Qualtrics) and had no associated completion incentive. This project was deemed exempt by the UCSD Institutional Review Board (exempt number 803916).

Statistical Analysis

The 5-point Likert-scale items on confidence in medical education skills were assessed as dichotomized variables to perform paired analyses. Participants who indicated they were moderately confident, somewhat confident, or not at all confident in their ability were compared to participants who indicated they were very confident or extremely confident in their ability. Multiple-choice questions assessing knowledge were analyzed as dichotomized variables (correct vs. incorrect). Differences were assessed using paired, two-tailed *t* tests. We performed descriptive statistics for the faculty and peer evaluations of the fellows' chalk talks during the practice session. Stata/IC version 28.0 (StataCorp) was utilized for statistical analyses, with $p < .05$ considered significant.

Results

Pre- and Postworkshop Outcomes

Thirty-three fellows completed the preworkshop survey, and 18 completed both the pre- and postworkshop surveys (54% response rate). Only data from participants who completed both the pre- and postworkshop surveys were included in the analysis. Respondents represented 12 different medical and surgical adult and pediatric subspecialties and ranged from postgraduate year (PGY) 4 to PGY 8 with respect to length of training. Thirteen respondents (72%) were female. Fellows' self-reported confidence across all medical education skills significantly improved following the workshop (Figure 1). Fellows reported improved confidence in their ability to create a chalk talk (22% vs. 83%, $p < .001$), deliver a chalk talk (17% vs. 83%, $p < .001$), and develop well-written learning objectives (11% vs. 83%, $p < .001$). Fellows' knowledge about underlying principles related to effective chalk talk design also significantly improved following the workshop (67% vs. 89%, $p < .05$).

Practice Session Chalk Talk Evaluations

Peer fellows and faculty completed 38 chalk talk feedback forms providing formative feedback to 24 fellows who presented chalk talks during the practice session (Figure 2). Most evaluations reported that fellows' chalk talks were delivered within a reasonable amount of time (5-15 minutes) for the clinical setting (95%, 36 of 38). Fellows' chalk talks were rated highly (maximum score of 2), receiving a score of 2 in domains of content suitability (95%, 36 of 38), chalk talk delivery (90%, 34 of 38), chalk talk design (68%, 26 of 38), learning objectives (66%, 25 of 38), and active learning strategies/audience engagement (74%, 28 of 38).

Discussion

We created a medical education chalk talk workshop to teach fellows foundational principles of creating and delivering a chalk talk and developing learning objectives. This initial session was coupled with a second one that provided an opportunity for deliberate practice with peer and faculty feedback using a

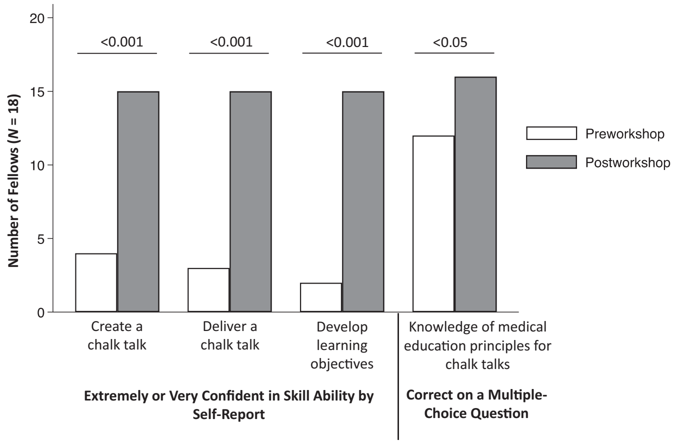

Figure 1. Pre- and postworkshop outcomes. The proportion of fellows reporting that they were extremely or very confident in their skill ability to create a chalk talk, deliver a chalk talk, and develop learning objectives significantly increased following the workshop compared to before it. The proportion of fellows correctly answering a multiple-choice question on medical education principles foundational to creating a chalk talk increased following the workshop, although this was not statistically significant.

Copyright © 2024 Soliman et al. This is an open-access publication distributed under the terms of the Creative Commons Attribution license.

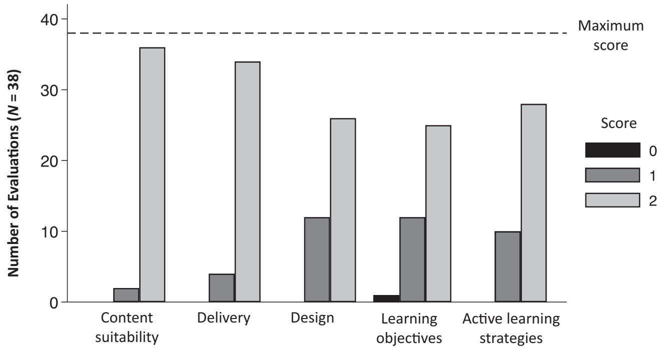

Figure 2. Practice session chalk talk evaluations across domains. Peers and faculty evaluated fellows' chalk talks, scoring each talk on a scale of 0-2 across five domains (content suitability, chalk talk delivery, chalk talk design, learning objectives, and use of active learning strategies) using a standardized evaluation tool. Chalk talks were scored highly (score of 2) across all domains.

structured feedback tool. Our educational intervention improved fellows' confidence in medical education skills, improved knowledge of education principles, and facilitated fellows developing chalk talks that were highly scored.

Employing formal training in teaching methods can improve teaching skills in the clinical setting. In an assessment of a 1-month clinician-educator track for 39 residents, Khan and colleagues implemented a curriculum that incorporated a session on giving a chalk talk followed by two 3-hour sessions practicing 15- to 20-minute chalk talks with resident and faculty feedback. Although there were few details about the content and curriculum of the chalk talk session, they reported an increase in the mean self-rated level of confidence (3.31, $SD = 0.04$, vs. 4.29, $SD = 0.32$; $p < .005$) after completion of the curriculum, as well as improved ability to plan and deliver an effective chalk talk ($p < .05$).[12] We observed similar trends but additionally demonstrated improvement in objective outcomes in fund of knowledge and evaluations of delivered chalk talks. We also provided detailed instructions on how to teach medical educators the skills and knowledge to develop and deliver chalk talks for the clinical setting in an efficient, two-part workshop. Overall, these findings suggest that the incorporation of a chalk talk workshop within a course on medical education can effectively develop clinical teaching skills among fellows.

Our workshop differs from many previously described fellow-as-teacher curricula. In a retrospective analysis of 44 trainees in a clinician-educator course that included dedicated sessions on presentation skills and projected visuals, Rosenbaum and colleagues revealed improvement in teaching skills as a result of a session on fellow mini-lectures (mean pretest score 2.60, $SD = 0.24$, vs. mean posttest score 3.10, $SD = 0.36$; $p = .001$).[13] Similarly, clinician-educator tracks and programs have utilized formative assessments of fellows' lectures in their curricula.[14] However, these sessions and evaluations of lectures have been designed to improve lecture PowerPoint slides and presentation skills. In contrast, we focused on a more specific type of teaching (chalk talks for the clinical setting) while simultaneously teaching generalizable skills such as constructing well-written learning objectives. Our chalk talk workshop was also unique in that it included the opportunity for deliberate practice with a standardized feedback form to facilitate constructive feedback from peers and faculty.

Our project had several important limitations. We used a subjective pre- and postworkshop self-evaluation of participant confidence, which may not reflect actual skills or ability. Our survey response rate of 54% may have introduced nonresponse bias in our results. We did not perform a pre- and postcourse evaluation of participant chalk talks, which would have allowed for the measurement of differences in teaching skills based on chalk talk performance. In addition, we were unable to assess the impact of the chalk talk session on fellows' teaching in the clinical setting, which could have resulted in different outcomes than the prepared standardized session delivered to faculty and peers. Our project included only fellow trainees and thus may lack generalizability to other groups of medical educators. That said, in conjunction with published literature, we predict that resident trainees as well as early career faculty would benefit from this workshop.

In summary, we have demonstrated the utility of incorporating a chalk talk workshop in a medical education course for subspecialty fellows. The benefits of chalk talks are multifactorial and include the ability to teach high-yield and clinically relevant topics by presenting focused information in a manner that allows flexibility, active learning, and engagement with material proximal to patient care.[6] In addition, chalk talks provide the opportunity to navigate clinical reasoning and decision-making with the educator in real time. Our resource adds to the growing literature on the use of chalk talks in medical education by demonstrating the effectiveness of a chalk talk workshop for clinical trainees. Our two-part workshop can be easily and efficiently implemented in a variety of training and educational development settings and thus may be broadly applied to other groups of medical educators.

Appendices

A. Chalk Talk Presentation.pptx

B. Assignment Instructions.docx

Copyright © 2024 Soliman et al. This is an open-access publication distributed under the terms of the Creative Commons Attribution license.

C. Resources on Creating Chalk Talks.docx

D. Feedback and Evaluation Tool.docx

E. Pre- and Postworkshop Survey.docx

All appendices are peer reviewed as integral parts of the Original Publication.

Shady I. Soliman, MD, MS: First-Year Resident, Department of Otolaryngology, University of California, Los Angeles, David Geffen School of Medicine

William McGuire, MD: Assistant Professor of Medicine, Department of Medicine, University of California, San Diego, School of Medicine

Tricia Santos, MD: Professor of Medicine, Department of Medicine, University of California, San Diego, School of Medicine

Charlie Goldberg, MD, MS: Professor of Medicine, Department of Medicine, University of California, San Diego, School of Medicine

Charles Coffey, MD: Professor of Surgery, Department of Otolaryngology, University of California, San Diego, School of Medicine

Darcy Wooten, MD, MS: Professor of Medicine, Department of Medicine, University of California, San Diego, School of Medicine

Disclosures

None to report.

Funding/Support

None to report.

Ethical Approval

The University of California, San Diego, Institutional Review Board deemed further review of this project not necessary.

References

1. Accreditation Council for Graduate Medical Education. *Program Director's Guide to the Common Program Requirements*. Accreditation Council for Graduate Medical Education; 2022.
2. Chen DC, Miloslavsky EM, Winn AS, McSparron JI. Fellow as Clinical Teacher (FACT) curriculum: improving fellows' teaching skills during inpatient consultation. *MedEdPORTAL*. 2018; 14:10728. https://doi.org/10.15766/mep_2374-8265.10728
3. McSparron JI, Huang GC, Miloslavsky EM. Developing internal medicine subspecialty fellows' teaching skills: a needs assessment. *BMC Medical Education*. 2018;18:221. https://doi.org/10.1186/s12909-018-1283-2
4. Kesselheim JC, Cassel CK. Service: an essential component of graduate medical education. *N Engl J Med*. 2013;368(6):500-501. https://doi.org/10.1056/NEJMp1214850
5. Miloslavsky EM, Boyer D, Winn AS, Stafford DEJ, McSparron JI. Fellows as teachers: raising the educational bar. *Ann Am Thorac Soc*. 2016;13(4):465-468. https://doi.org/10.1513/AnnalsATS.201601-026PS
6. Stetson GV, Dhaliwal G. Chalk talks in the clinical learning environment. *Acad Med*. 2023;98(4):534. https://doi.org/10.1097/ACM.0000000000004848
7. Singh N, Phoon CKL. Not yet a dinosaur: the chalk talk. *Adv Physiol Educ*. 2021;45(1):61-66. https://doi.org/10.1152/advan.00126.2020
8. Pitt MB, Orlander JD. Bringing mini-chalk talks to the bedside to enhance clinical teaching. *Med Educ Online*. 2017;22(1):1-7. https://doi.org/10.1080/10872981.2017.1264120
9. Morris GA, Vaz K, Russo J, et al. Implementing mini-chalk talks to enhance teaching and learning. *Clin Teach*. 2021;18(6):577-582. https://doi.org/10.1111/tct.13394
10. Krathwohl DR. A revision of Bloom's taxonomy: an overview. *Theory Pract*. 2002;41(4):212-218. https://doi.org/10.1207/s15430421tip4104_2
11. Artino AR Jr, La Rochelle JS, Dezee KJ, Gehlbach H. Developing questionnaires for educational research: AMEE Guide no. 87. *Med Teach*. 2014;36(6):463-474. https://doi.org/10.3109/0142159X.2014.889814
12. Kahn MJ, Estrada CA, Willett LL, Kraemer RR. Creating clinician educators: evaluation of a one-month medical education curriculum for senior residents. *Am J Med Sci*. 2021;362(6): 606-611. https://doi.org/10.1016/j.amjms.2021.06.010
13. Rosenbaum ME, Rowat JA, Ferguson KJ, et al. Developing future faculty: a program targeting internal medicine fellows' teaching skills. *J Grad Med Educ*. 2011;3(3):302-308. https://doi.org/10.4300/JGME-D-10-00109.1
14. Adamson R, Goodman RB, Kritek P, Luks AM, Tonelli MR, Benditt J. Training the teachers: the clinician-educator track of the University of Washington pulmonary and critical care medicine fellowship program. *Ann Am Thorac Soc*. 2015;12(4):480-485. https://doi.org/10.1513/AnnalsATS.201501-032OT

Received: July 21, 2023
Accepted: November 20, 2023
Published: March 5, 2024

Copyright © 2024 Soliman et al. This is an open-access publication distributed under the terms of the Creative Commons Attribution license.
